# What Is the Willingness to Receive Vaccination Against COVID-19 Among the Elderly in Italy? Data From the PASSI d'Argento Surveillance System

**DOI:** 10.3389/fpubh.2021.736976

**Published:** 2021-11-05

**Authors:** Benedetta Contoli, Valentina Possenti, Valentina Minardi, Nancy J. Binkin, Mauro Ramigni, Giuliano Carrozzi, Maria Masocco

**Affiliations:** ^1^National Center for Disease Prevention and Health Promotion, Italian National Institute of Health, Rome, Italy; ^2^Wertheim School of Public Health, University of California, San Diego, San Diego, CA, United States; ^3^Prevention Department, Local Health Authority 2, Treviso, Italy; ^4^Department of Public Health, Local Health Unit of Modena, Modena, Italy

**Keywords:** vaccine uptake, hesitancy, elderly, COVID-19, behavioral surveillance system, seasonal flu, public health

## Abstract

**Introduction:** Italy was one of the earliest countries to experience a severe COVID-19 epidemic and vaccinating the elderly, who constitute 23% of the population and have experienced the highest mortality rates, is a top priority. Estimating prevalences and understanding risk factors for COVID-19 vaccine hesitancy or refusal are important for development of targeted interventions.

**Methods:** We used data from a specially developed COVID-19 module of PASSI D'Argento, an ongoing surveillance system of residents 65+ years of age to measure the prevalence and identify risk factors for hesitancy and refusal to receive the COVID-19 vaccine. We calculated multinomial regression relative risk ratios to examine the association between demographic characteristics, health status, COVID-19 attitudes and experiences and likely vaccine hesitancy and refusal.

**Results:** Of the 1876 respondents, 55% reported they would accept vaccination and 16% would likely refuse; the remaining 29% were categorized as hesitant. Compared with the *acceptance* group, we identified several risk factors in common between the *hesitancy* group and the *refusal* group, including not having received vaccination against influenza during the previous flu season (hesitancy: RRR = 2.0; 95% CI 1.4–2.9; refusal: RRR = 12.1; 95%CI 7.6–19.4) and lower risk of having had a death from COVID-19 among family or friends (hesitancy: RRR = 4.8; 95%CI 2.0–11.4; refusal: RRR = 15.4; 95%CI 3.7–64.5). The *hesitancy* group was significantly more likely being worried and they did not know if consequences of the disease would be serious for them.

**Conclusion:** Our findings show the importance of establishing and maintaining active contact between the preventive services, primary care providers and the population because trust is difficult to establish during an emergency like the COVID-19 pandemic. Italian public health is based on a capillary network of general practitioners and having them reach out to their patients who have not previously received influenza vaccine may be a useful strategy for targeting efforts to further encourage uptake of COVID-19 vaccination.

## Introduction

Italy was one of the earliest countries to experience a severe COVID-19 epidemic (1). As of mid-May 2021, the country reported over 4.1 million cases and over 120,000 deaths (2). The elderly in Italy have been disproportionately affected, with persons over 60 years accounting for approximately more than a quarter of reported cases and nearly 95% of the deaths.

While measures such as masking and social distancing can reduce spread of the virus, high coverage with COVID-19 vaccines is widely regarded as essential if the epidemic is to be controlled (3). However, given the emergence of variants and other factors such as increasing social contact and the long-lasting pandemic globally, achieving and sustaining high levels of vaccination in the population is actually the greatest challenge in most countries.

The vaccines against COVID-19 became available in Italy at the end of December 2020 and were administered following a specific national vaccination plan (4). As in many other countries, vaccinating the elderly, who represent 23% of Italy's population and have the highest COVID-19 mortality rates, is a top priority. Italy has an extensive preventive health network where vaccines are provided free of charge, but in the past, uptake of seasonal influenza vaccines has not been optimal. Coverage among the elderly in 2019–20 was estimated at 55%, considerably below the minimal national objective of 75% coverage and the optimal objective of 85% (5). There is a gradient by age group in the estimates of the influenza vaccination coverage, from 47% in the 65–74 years age group to 63% in those 75–84 years and 71% in those 85 and older.

Understanding the extent to which the elderly may be hesitant or refuse to be vaccinated against COVID-19 and factors associated with these attitudes is important for the development of public health interventions both at an individual and population level. To measure the prevalence and identify risk factors for hesitancy and refusal toward the COVID-19 vaccine and, then to assess changes over time among the elderly in Italy, we used data from PASSI D'Argento, an ongoing national behavioral risk factor surveillance system of Italian residents 65 years of age and over. This system, which began as a series of periodic cross-sectional surveys conducted between 2009 and 2015, became a surveillance system in 2016 and collects continuously data on health status, quality of life, and health behaviors associated with the most common chronic diseases, participation in society and employment, independent living, safety and life environment (6). In August 2020, a special COVID module was added to the standard questionnaire to investigate several COVID-related patterns such as: risk perceptions, experience with the disease, its impact on economic conditions, emotional well-being, and accessibility to care, mask use, trust in the ability of the healthcare system to manage the emergency, and likely willingness to be vaccinated (7).

## Methods

### Sampling and Data Collection Methods

Each participating region or autonomous province contains local health units, which cover populations ranging from 150,000 to more than a half-million residents (8). All residents are registered with their local health unit, which provides both preventive and curative services. At intervals ranging from 3 to 6 months, the health units select a sample of persons ages 65 and older to interview in PASSI D'Argento from their register that is stratified by gender and age (65–74, 75−84 and ≥85 years of age). Persons whose primary residence is in another region, who do not have a valid telephone number, who are currently hospitalized or in long-term care, nursing homes or prisons are excluded from the sample. Those who do not speak Italian are also excluded except in the autonomous province of Bolzano, where interviewees have the option of being interviewed in German. Each person selected receives a letter from their general practitioner informing them that they will be contacted. Prior to the COVID-19 pandemic, trained personnel from each health unit's social and health services interviewed those selected by phone or, for those with hearing or other problems, they conducted the interview face-to-face. However, because of COVID-19, in-person, interviews were conducted only over the telephone. Further methodological details can be found elsewhere (6). Over the years, response rate has remained very high and, was 84% in 2020 according to the parameters indicated by the guidelines of the American Association for Public Opinion Research (9).

As of January 31, 2021, a total of 5063 interviews had been gathered for the period from January 2020 to December 2020. However, 2681 interviews finalized after the COVID-19 module was introduced in August 2020 were eligible for inclusion in the analysis.

### Study Variables and Definitions

The major outcome variable for this study, willingness to be vaccinated against COVID-19, was assessed by asking respondents:” If a vaccine against COVID-19 were available, would you be willing to be vaccinated?” Four answer options were possible: “Definitely yes,” “Probably yes,” “Probably no,” “Definitely no.” Additionally, the PASSI d'Argento interviewers were able to record the following spontaneous answers: “I have already had COVID-19” and “I don't know.” They also recorded instances where the interviewee was unable or unwilling to respond to the question.

**Figure 1:**
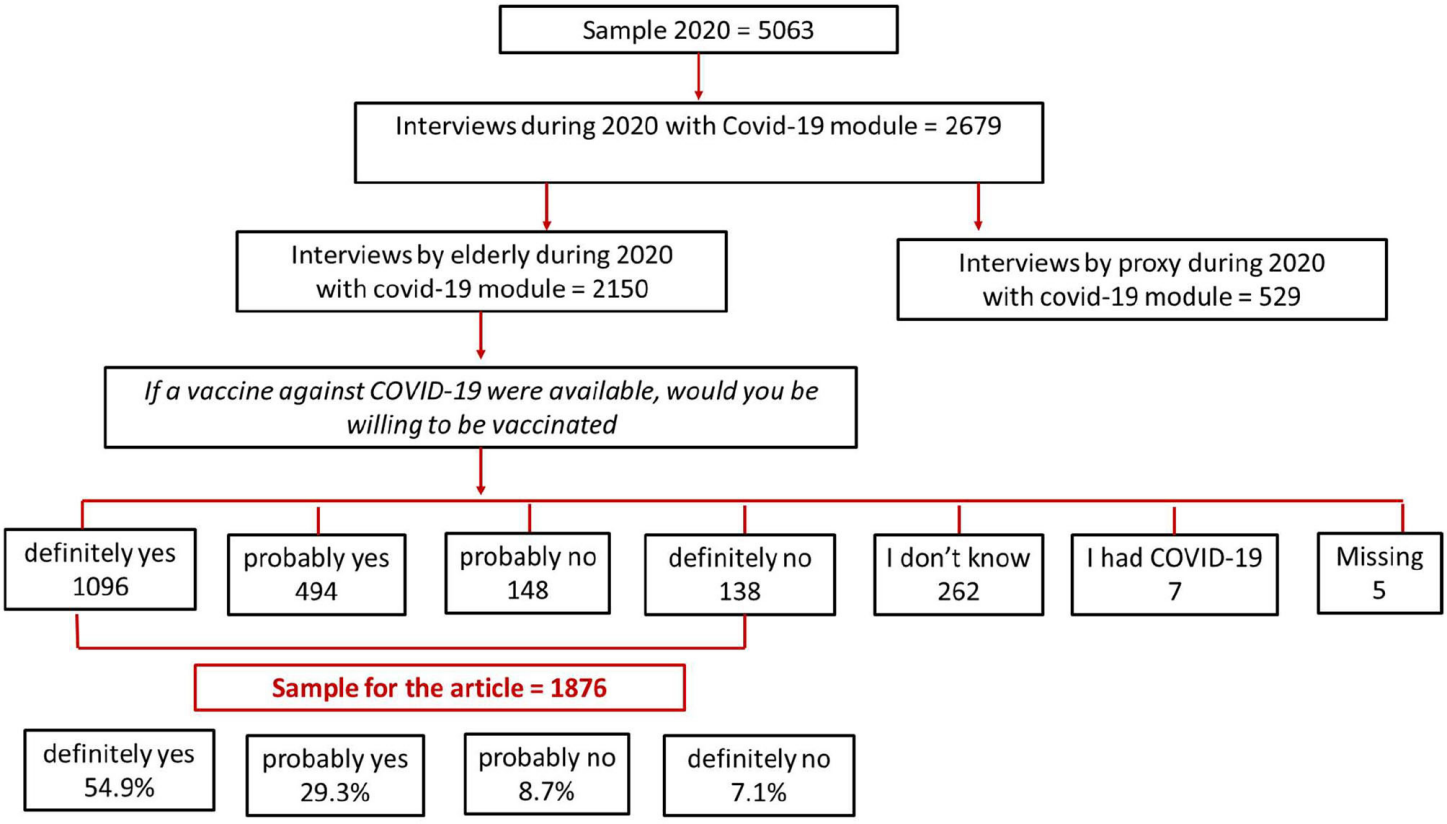
Flow chart for selected sample. Passi D'Argento, Italy, August-December 2020.

Figure 1 shows the number of persons excluded and reasons for exclusion from the analysis. We eliminated those who had already had COVID-19, and who were unable or unwilling to respond to the question regarding vaccination. The “I don't know” category, which accounted for 262 responses, was eliminated from the analysis after extensive investigation into the patterns of response by health unit and interviewer revealed clustering, suggesting that this response may in part have represented a lack of probing on the part of the interviewer rather than a reflection of what the interviewee responded. We then excluded five questionnaires with missing data on vaccine attitude. Overall, 1,876 persons were included in the analysis.

We then examined the characteristics of those responding “definitely yes,” “probably yes,” “probably no,” and “definitely no” to the COVID-19 vaccination question. Pearson's chi square was used to analyze differences between the four groups. We found that there were no statistically significant differences between those responding “probably no” and “definitely no” with respect to gender (χ^2^ = 0.58), educational level (χ^2^ = 0.13), and area of residence (χ^2^ = 0.27). For the subsequent analyses, we therefore created three groups: acceptance (“definitely yes”), hesitancy (“probably yes”), and refusal (“probably no” and “definitely no”).

The independent sociodemographic variables considered in our analyses included the following:

- Age: 65–74, 75–84, and 85+ years.
- Gender: male and female.
- Education: low (none or elementary school), high (middle school or higher).
- Economic difficulties (economic difficulties in making ends meet by the available household financial resources): none, some, many.
- Social isolation: yes, no.
- Degree of urbanization: high/very high, medium low (National Institute of Statistics; ISTAT) (10).
- Area of residence: North Center, South (National Institute of Statistics; ISTAT) (11).

The health status characteristics examined included the following:

- Influenza vaccine during the previous year: yes, no.
- Disability: Having trouble performing one or more ADL; yes/no.
- Frailty: Having problems performing two or more Instrumental Activities of Daily Living (IADL), but autonomous in all Activities of Daily Living (ADL); yes/no.
- Chronic diseases: information retrieved by individual yes/no answering questions on self-reported presence of diabetes, kidney failure, chronic bronchitis, emphysema, asthma, respiratory insufficiency, stroke myocardial infarction, coronary and other heart diseases, tumors, chronic liver disease or cirrhosis; none, at least one.

Finally, attitudes and experiences related to COVID-19:

- Perceived risk of infection to themselves or to the own family within the next 3 months: high/somewhat high, low or none, don't know.
- Perceived risk of serious own health outcomes if infected: serious (serious or very serious), not serious (fairly serious or none), don't know.
- Trust in local health unit to manage the COVID-19 situation: high/somewhat high; low or none.
- Bereavements in family or friends due to the coronavirus: yes, no.
- Being concerned about the current situation: yes (very worried, somewhat worried), no (not very worried; not worried at all).

### Data Analysis

We used univariate logistic analysis to test the association of sociodemographic, health, and COVID-19 experience variables with the vaccination attitude outcome. The reference population for all analyses was the acceptance group. We then conducted a multinomial regression model that excluded variables with *p* > 0.20 in the univariate analysis. Although they did not meet the *p*-value cutoff, we also included the presence of one or more chronic diseases and disability in the final models.

The outcome variable in the multinomial regression model was coded so that the *acceptance* group was compared with the *hesitancy* and *refusal* groups. Multinomial regression coefficients were exponentiated and are presented as relative risk ratios (RRR) with corresponding 95% confidence intervals (CI). The RRRs compare the risk of an outcome (in this case hesitancy and refusal to vaccinate against COVID-19) among one group compared with a reference group (e.g., females compared with males, the latter serving as the referent group) (12). All data were analyzed using STATA, version 16.0 (Stata Corp, College Station, TX, USA) (13).

## Results

Sociodemographic and health status characteristics of the 1,876 persons interviewed who were included in the analysis are shown in Table 1. Over half (52%) were in the 65–74 years age group, and there were more females than males (54 vs. 46%). More than a third (36%) had at most attended elementary school, in keeping with the lack of enforced obligatory schooling in many parts of Italy before the 1950s. The majority did not report financial difficulties and 7% live in social isolation conditions. Although 42% had at least one non-communicable chronic disease, a minority suffered from one or more disabilities (7%) or frailty (12%). Overall, 68% reported having received the influenza vaccine in the last 12 months, with values ranging from 61% for those 65 to 74 years to 80% in those 85 years and older.

**Table 1:** Characteristics of Italian elderly overall and by willingness to vaccinate against COVID-19, Passi d'Argento, August-December 2020.

**Characteristic**				**Subgroups by willingness to vaccinate against COVID-19**					
		**All**		**Acceptance (*N* = 1,096)**		**Hesitancy (*N* = 484)**		**Refusal (*N* = 286)**	
		***N***	**%**	**%**	**IC 95%**	**%**	**IC 95%**	**%**	**IC 95%**
**Total**		1,876	–	54.9	(51.9–57.9)	29.3	(26.5–32.2)	15.8	(13.5–18.5)
Gender	Male	856	46.4	49.5	(46.7–52.2)	47	(42.1–52.0)	34.5	(27.4–42.5)
	Female	1,019	53.6	50.6	(47.8–53.3)	53	(48–57.9)	65.5	(57.6–72.6)
Age group	65–74	925	52	52.1	(49.4–54.8)	47	(42.2–52.0)	60.7	(52.7–68.2)
	75–84	746	37.4	37.5	(34.9–40.2)	40.6	(35.6–45.8)	30.9	(23.9–39.0)
	85+	204	10.6	10.4	(9.0–12.0)	12.4	(9.5–16.0)	8.4	(5.7–12.1)
Educational level	Low	677	35.9	32.2	(28.4–36.3)	44.7	(38.7–50.9)	32.1	(24.9–40.2)
	High	1,189	64.1	67.8	(63.7–71.6)	55.3	(49.1–61.3)	67.9	(59.8–75.1)
Economic difficulties	Many	109	5.3	4.3	(2.9–6.2)	6.3	(3.0–12.6)	6.7	(4.2–10.7)
	Some	376	22.5	18.8	(15.9–22.0)	28.7	(22.5–35.7)	24.5	(17.5–33.1)
	None	1,368	72.2	77	(73.6–80.1)	65.1	(59–70.7)	68.8	(60.3–76.2)
Population density	High/very high	560	34.5	34.1	(29.9–38.6)	31.3	(25.7–37.5)	42	(33.5–51.1)
	Moderate	644	37.3	36.8	(32.9–40.9)	39.8	(34.1–45.8)	34.8	(27.5–43.0)
	Low	670	28.1	29.1	(26.1–32.3)	29	(24.1–34.4)	23.1	(17.4–30.1)
Geographic area of residence	North	1,155	61	66.8	(64.2–69.4)	50.9	(45.9–55.9)	59.6	(51.3–67.3)
	Center	188	11.2	9.5	(7.9–11.3)	13.2	(9.8–17.5)	13.7	(9.2–19.9)
	South	532	27.8	23.7	(21.4–26.0)	35.9	(31.1–41.0)	26.8	(19.8–35.1)
Seasonal flu vaccination uptake	Yes	1,263	67.5	80.2	(76.2–83.7)	66.7	(61.2–71.9)	24.8	(18.9–31.8)
	No	602	32.5	19.8	(16.4–23.8)	33.3	(28.1–38.8)	75.2	(68.2–81.1)
Disabilities	Yes	100	7.4	6.5	(4.8–8.9)	11.5	(8.2–15.9)	3.2	(1.8–5.6)
	No	1,739	92.6	93.5	(91.1–95.2)	88.5	(84.1–91.9)	96.8	(94.4–98.2)
Frailty	Yes	218	12.3	10.4	(8.3–12.9)	15.9	(11.6–21.4)	12.3	(7.0–20.9)
	No	1,621	87.7	89.6	(87.1–91.7)	84.1	(78.6–88.4)	87.7	(79.1–93)
Isolation	Yes	125	6.68	5.7	(4.1–7.9)	9.9	(5.7–16.6)	4	(2.4–6.7)
	No	1,744	93.32	94.3	(92.1–95.9)	90.1	(83.4–94.3)	96	(93.3–97.6)
Non-communicable chronic diseases	None	827	42.1	38.6	(34.5–42.9)	39.4	(34.0–45.2)	59.4	(50.8–67.5)
	At least one	1,046	57.9	61.4	(57.1–65.6)	60.6	(54.8–66.1)	40.6	(32.5–49.2)
**COVID-19 attitudes and experience**									
Probability of infection of SarsCov2	High/somewhat high	642	39.8	40.8	(36.7–45.1)	45.1	(39.4–50.8)	26.6	(19.1–35.7)
	Low or none	819	42.5	40.9	(37.4–44.5)	44.5	(34.1–45.0)	53.7	(45.5–61.8)
	I don't know	414	17.7	18.3	(15.1–21.9)	21.9	(12.3–19.4)	19.7	(14.5–26.1)
Consequences of SarsCov2	Serious	1,052	61.6	65.7	(62.3–69.0)	69	(57.3–67.8)	45.2	(36.6–54.0)
	Not serious	354	19.3	17.1	(14.7–19.8)	19.8	(12.8–20.5)	32.2	(24.0–41.8)
	I don't know	462	19.2	17.2	(14.8–19.9)	19.9	(17.1–25.6)	22.6	(17.7–28.4)
Reported intrusive thoughts	Yes	346	31.1	31	(27.0–35.3)	35.3	(32.9–44.4)	18	(11.0–28.0)
	No	1,021	68.9	69	(64.7–73)	73	(55.6–67.1)	82	(72.1–89.0)
Reported being worried	Yes	1,476	81.6	82.2	(79.3–84.7)	84.7	(86.5–91.7)	64.9	(56.9–72.1)
	No	385	18.4	17.8	(15.3–20.7)	20.7	(8.3–13.5)	35.1	(27.9–43.1)
Trust in local health unit management	High/somewhat high	1,053	76.8	80.5	(76.3–84.1)	84.1	(66.5–79.6)	69.6	(60.1–77.6)
	Low or none	345	23.3	19.5	(15.9–23.7)	23.7	(20.4–33.5)	30.5	(22.4–39.9)
COVID-19 cases in family, friends, or colleagues	Yes	484	32.7	34	(30.6–37.6)	37.6	(28.7–41.3)	24.9	(18.0–33.3)
	No	1,375	67.3	66	(62.4–69.4)	69.4	(58.8–71.3)	75.1	(66.7–82.0)
COVID-19 deaths in family or friends	Yes	80	7	10.9	(7.4–15.8)	15.8	(1.5–4.7)	1.1	(0.2–4.8)
	No	1,303	93	89.1	(84.2–92.6)	92.6	(95.3–98.5)	98.9	(95.2–99.8)

Experience with COVID-19, perceived risk, and expressed likelihood of getting the vaccine are shown in Table 1. A total of 40% thought it was likely or very likely that they or someone in their family would get COVID-19 in the next 3 months, and 62% was concerned that if they did get it, the own health consequences would be serious. Trust in their local health unit to manage the COVID-19 situation was high (77%). Most of those interviewed (82%) were worried or slightly worried about the current situation regarding COVID-19, and 31% had experienced intrusive thoughts. Nearly a third had had a relative, friend, or colleague who developed COVID-19, and 7% percent had experienced a death in a family member or close friend.

In response to the question, “*If a vaccine against COVID-19 were available, would you be willing to be vaccinated?,”* 55% replied “definitely yes,” 29% “probably yes,” 9% “probably no,” and 7% “definitely no.” Over the 4 months included in the study, which occurred before the introduction of the vaccine in Italy, the frequency of each response category remained relatively stable.

As noted in the methods section, the demographic characteristics of the “probably no” and “definitely no” were similar, and “probably no” and “definitely no” were merged to form a *refusal* group. The subsequent analysis is therefore based on three groups: *acceptance*, which consisted of those responding “definitely yes” (1,096; 55%), *hesitancy*, which consisted of those responding “probably yes” (494; 29%) and *refusal*, which included the combined “definitely no” and “probably no” (286; 16%). Characteristics associated with each of the three intention-to-vaccinate categories are shown in Table 1.

Results of the univariate and multinomial analyses of risk factors for *hesitancy* are shown in Table 2. All but three risk factors identified in the univariate analysis (economic difficulties, frailty, and disability) remained significant in the multinomial model, including lower education (RRR = 1.6; 95% CI 1.1–2.4, living in the center area of Italy (RRR = 1.7; 95% CI 1.1–2.7) and stating that they did not know if consequences of the disease would be serious for them (RRR = 1.9; 95% CI 1.3–2.9). The *hesitancy* group was less likely to be worried about the current situation (RRR = 0.6; 95% CI 0.4–0.9).

**Table 2:** Association between Sociodemographic and COVID-related risk factors and hesitancy or refusal to accept the COVID-19 vaccine among Italian elderly.

		**Univariate analysis**				**Multivariate analysis**			
		**Logistic model**		**Logistic model**		**Multinomial Regression model**			
**Characteristics**		**Hesitancy vs. acceptance**		**Refusal vs. acceptance**		**Hesitancy**		**Refusal**	
**Sociodemographic factors**		**ORs**	***95% CI***	***ORs***	***95% CI***	***RRRs***	***95% CI***	***RRRs***	***95% CI***
Gender *(ref male)*	Female	1.10	(0.84–1.45)	**1.86**	**(1.26–2.74)**	1.17	(0.86–1.59)	**1.83**	**(1.21–2.77)**
Age group *(ref 65–74)*	75–84	1.20	(0.88–1.63)	0.71	(0.46–1.08)	0.98	(0.69–1.37)	0.96	(0.57–1.61)
	85+	1.32	(0.86–2.02)	0.69	(0.42.1.15)	0.91	(0.56–1.49)	1.17	(0.54–2.57)
Educational level *(ref High)*	Low	**1.70**	**(1.23–2.34)**	0.99	(0.66–1.49)	**1.63**	**(1.11–2.39)**	1.48	(0.91–2.38)
Economic difficulties *(ref None)*	Some	**1.81**	**(1.24–2.63)**	1.46	(0.90–2.37)	1.3	(0.81–2.08)	1.48	(0.85–2.60)
	Many	1.72	(0.74–4.01)	1.76	(0.93–3.31)	1.57	(0.57–4.32)	2.31	(0.89–6.00)
Population density *(ref Low)*	Moderate	1.09	(0.78–1.51)	1.19	(0.78–1.82)	0.93	(0.62–1.39)	**1.71**	**(1.01–2.88)**
	High	0.92	(0.62–1.37)	1.55	(0.94–2.55)	1.05	(0.68–1.61)	**2.07**	**(1.16–3.71)**
Geographic area of residence *(ref North)*	Center	**1.82**	**(1.14–2.92)**	1.61	(0.90–2.89)	1.7	(1.07–2.68)	**2.14**	**(1.03–4.43)**
	South	**2.00**	**(1.45–2.73)**	**1.27**	**(0.81–2.00)**	1.55	(0.97–2.47)	1.58	(0.87–2.86)
Seasonal flu vaccination uptake *(ref Yes)*	No	**2.02**	**(1.45–2.81)**	**12.28**	**(8.15–18.50)**	**2.02**	**(1.41–2.89)**	**12.11**	**(7.57–19.37)**
Disabilities *(ref Yes)*	No	**1.85**	**(1.13–3.04)**	**0.47**	**(0.24–0.93)**	1.47	(0.85–2.53)	0.45	(0.15–1.38)
Frailty *(ref Yes)*	No	**1.63**	**(1.06–2.50)**	1.21	(0.61–2.40)	1.67	(0.95–2.94)	1.9	(0.94–3.81)
Isolation *(ref Yes)*	No	1.81	(0.91–3.58)	0.69	(0.36–1.31)	–	–	–	–
Non-communicable chronic diseases *(ref At least one)*	None	1.04	(0.76–1.40)	**2.33**	**(1.54–3.53)**	1.14	(0.81–1.60)	**1.65**	**(1.07–2.53)**
**COVID-19 attitudes and experiences**									
Probability of infection of SarsCov2 *(ref Very/somewhat High)*	Low or none	0.87	(0.64–1.20)	**2.02**	**(1.27–3.19)**	0.98	(0.67–1.44)	1.48	(0.86–2.56)
	I don't know	0.77	(0.51–1.16)	1.65	(0.94–2.91)	0.76	(0.46–1.24)	1.96	(0.94–4.06)
Consequences of SarsCov2 *(ref Serious)*	Not serious	1.00	(0.70–1.43)	**2.75**	**(1.71–4.41)**	1.27	(0.84–1.93)	**1.77**	**(1.00–3.13)**
	I don't know	1.28	(0.92–1.78)	**1.91**	**(1.29–2.83)**	**1.90**	**(1.25–2.90)**	1.53	(0.85–2.75)
Reported being worried *(ref Yes)*	No	**0.55**	**(0.39–0.77)**	**2.50**	**(1.69–3.70)**	**0.62**	**(0.41–0.95)**	**2.65**	**(1.65–4.27)**
Trust in management capacity by local health unit *(ref High/somewhat High)*	Low or none	1.48	(0.97–2.26)	**1.80**	**(1.16–2.81)**	1.55	(0.99–2.41)	1.73	(0.97–3.08)
	I don't know	1.19	(0.86–1.64)	1.32	(0.86–2.03)	1.28	(0.88–1.86)	1.51	(0.86–2.63)
COVID-19 cases among family, friends, or colleagues *(ref No)*	Yes	0.97	(0.71–1.34)	**1.56**	**(1.02–2.37)**	1.04	(0.73–1.50)	1.18	(0.75–1.88)
COVID-19 deaths among family or friends *(ref Yes)*	No	**4.51**	**(2.17–9.34)**	**11.35**	**(2.34–55.14)**	**4.83**	**(2.05–11.36)**	**15.36**	**(3.66–64.46)**

*Passi d'Argento, August-December 2020. Crude Odds ratios (ORs) by univariate logistic models and adjusted Relative Risk Ratios (RRRs) by Multinomial Regression model, with relative 95% Confidence Intervals (95%CI)*.

*Values in bold show statistically significant ORs (in univariate analysis) and RRRs (in multinomial regression)*.

The strongest associations, however, were not having experienced a death among family or friends (RRR = 4.8; 95% CI 2.0–11.4) and not having received influenza vaccine in the past year (RRR = 2.0; 95% CI 1.4–2.9).

Results of the univariate and multinomial risk factors for refusal are also shown in Table 2. They overlapped with risk factors seen in of the hesitancy group. However, they differed in magnitude, especially not having experienced a death among family or friends (RRR = 15.4; 95% CI 3.7–64.5) and not having received influenza vaccine in the past year (RRR = 12.1; 95% CI 7.6–19.37). Factors that emerged in the *refusa*l group that were not present in the analysis of the *hesitancy* group included being female (RRR = 1.8; 95% CI 1.2–2.8), and living in high/very high- (RRR = 1.7; 95% CI 1.0–2.9) and moderate- density areas (RRR = 2.1; 95% CI 1.2–3.7), not having chronic diseases (RRR = 1.65; 95% CI 1.1–2.5) and not being worried about the current situation (RRR = 2.7; 95% CI 1.7–4.3). Additional factors that were significant in the univariate analysis but did not reach the threshold for significance in the multinomial analysis included perceived risk of becoming infected and experiencing serious complications if infected, absence of a disability, trust in the health care system to manage the situation, and COVID-19 infections in family or friends.

## Discussion

In our study, 55% of the elderly population was highly willing to be vaccinated, and a minority (16%) would likely refuse the vaccine, with the remaining 29% categorized as hesitant. These values did not change substantially over the 4-month study period, which occurred prior to the initiation of vaccination efforts in Italy at the end of December.

At the time of this writing in May 2021, extensive efforts have been made to reach the Italian population aged 70 years and older. An estimated 13% of those over 80 years and 33% of those between 70 and 79 years remain unvaccinated (14) suggesting that many of those who were initially hesitant or unwilling have ultimately been vaccinated. Surveillance data from PASSI d'Argento will permit an examination of how responses changed over time and whether those who are currently not vaccinated simply lack access, are still hesitant or even more fixed in their attitudes about not being vaccinated.

Understanding factors involved in the decision-making process may nonetheless be useful in increasing vaccination against COVID-19 in the elderly as well as improving current influenza vaccination coverage and in ensuring high update of vaccines should further pandemics occur.

The strongest risk factors for both hesitancy and refusal were not having experienced a death among a relative or friend and not having gotten the flu vaccine previously, although the magnitude of the associations was far greater among the *refusal* group. Those who had not experienced a loss were 4.8 times more likely to be hesitant and 15 times more likely to refuse vaccination. Perhaps more importantly, however, from the public health intervention point of view, is that the hesitant group was twice as likely and the refusal group 12 times more likely not to have gotten the flu vaccination during the previous season: only 20% of the *acceptance* group had not been vaccinated, compared with 33% among the *hesitant* and 75% among the *refusal* group. The refusal group also had a notably lower level of chronic diseases and were less likely to perceive COVID-19 as a serious illness. These findings, combined with those regarding trust in the system to handle the problem, suggest ongoing issues with the ability of the local health units and providers to convince their elderly populations of the importance of vaccination.

Valid comparisons with other studies on COVID-19 vaccination attitudes are difficult to make. First, definitions of hesitancy and refusal have not been consistent (15). Second, risk factors vary from country to country because of different cultural and organizational issues (15, 16); for example, in some, older age is associated with increased hesitancy, while in others, it is associated with decreased hesitancy. Third, few studies have specifically examined attitudes of the elderly toward the COVID-19 vaccination or had sample sizes sufficient to stratify predictors by age. Finally, there appear to be a wide variety of social, environmental, and psychological factors that influence vaccine decisions, as well as issues of trust in the government and the medical system and political factors and perceptions of threat (15–19). The situation is highly dynamic, as indicated by the rapidly changing attitudes as greater experience is gained with vaccine, first from the trials and now from widespread vaccination also depending on different vaccine technology and manufacture (15, 17, 18), and data acquired at different points in time are difficult to compare.

A consistent finding from several studies, however, has been the association between influenza vaccine practices and potential COVID-19 vaccine hesitancy or refusal in Italy (17) and in the US (20–22). It is also consistent with previous literature on prior influenza vaccines and the willingness to be vaccinated during other pandemics (23). Whether some of the reluctance in other settings has been due to concerns about the influenza vaccine and its effectiveness, it also appears to be influenced by trust in the health care system, which is more difficult to modify.

The finding regarding influenza vaccine may nonetheless help in targeting additional efforts to those who are currently reluctant or unwilling to be vaccinated. In Italy, each person is assigned a general practitioner, and many of the elderly have had longstanding relationships with these physicians and trust in their advice. Centralized records are kept in each local health unit of the physician to whom each person is assigned as well as on their vaccination records, which could be used as a way of identifying those who might benefit from benefit from an individual letter or call from their general practitioner in deciding to get the COVID-19 vaccine. Supporting the general practitioners in their role as educators may be an important means of providing older individuals with adequate and accessible information and motivation regarding vaccination.

Our finding concerning greater hesitancy and reluctance to vaccinate among those who reported not having family members or friends who became ill or who died raises interesting questions about the future of COVID-19 vaccine efforts over time as the number of cases deaths due to COVID-19 in Italy have begun to decrease dramatically and the immediacy of severe illness and death family members or friends may no longer be an important motivator of vaccination.

International studies have used a wide variety of definitions of what constitutes hesitancy and refusal based on the way the questions have been asked and the classifications of responses by investigators (15). Ours used a less conventional approach of treating those who said “probably yes” as our hesitancy group and the “probably no” and definitely no” as the refusal group. We found in our preliminary analyses that those who said they probably would not be vaccinated aligned most closely with those who said they would not get the vaccine, while those who responded that they probably would be vaccinated were the closest equivalent of a hesitancy group in our study. This finding may have been explained by social desirability bias that could have arisen because the interviews are done by local health staff from the interviewee's health unit of reference. Those interviewed may have been reluctant to admit to someone from their local unit that they probably would not get the vaccine and instead chose to respond that they probably would.

As has been demonstrated elsewhere (17, 18, 24), the situation is highly dynamic, and having an agile system that can monitor changing attitudes will be important to altering the messaging and identifying changes in the characteristics of those hesitant or unwilling to be vaccinated. Behavioral risk factors systems such as PASSI d'Argento have considerable capacity to produce such data, especially if data analysis is decentralized to look at the situation at regional or sub-regional level.

Our study has some limitations. First, it represents a window of time prior to the widespread introduction of the vaccine. As increasingly large numbers of people are vaccinated in Italy and elsewhere, vaccine acceptance has increased, and the characteristics of those who are hesitant or likely to refuse may have changed. Data collection for PASSI d'Argento is ongoing, and it will be important to monitor future trends and reassess factors associated with hesitancy and refusal.

Another limitation is the lack of information on the reasons behind hesitancy or reluctance to be vaccinated among our study participants. However, because of the multi-purpose nature of PASSI d'Argento as a surveillance system for several health issues of the elderly, the number of questions on each topic is limited and other methods will be needed to collect more in-depth information.

In conclusion, we found that the majority of Italian elderly expressed interest in being vaccinated, and that refusal levels were likely to be relatively low, which has been borne out in the country's experience after the vaccine was introduced. Understanding the characteristics of those who are hesitant to be vaccinated and those who refuse may aid in the tailoring of health messages that take into account perceptions, barriers, and concerns. Identifying those who have not been immunized against influenza may be a relatively easy way of identifying those who may benefit from individualized messages from their personal health providers.

## Data Availability Statement

The raw data supporting the conclusions of this article will be made available by the authors, without undue reservation.

## Ethics Statement

The studies involving human participants were reviewed and approved by the Ethics Committee of Italian National Institute of Health. Oral informed consent was obtained from the participants of the study.

## Author Contributions

BC contributed to designing the study, data collection and analysis, wrote the first draft of the article, and contributed to the different versions of the manuscript. VP contributed to the data dissemination and wrote some sections of the article. VM contributed to the data collection and analysis and revised the final version of the manuscript. NB gave a substantial contribution to the interpretation of the data and revised the final version of the manuscript. MR gave an important contribution to the literature review and revised the discussion section. GC contributed to the final version of the manuscript and the data dissemination. MM revised the whole manuscript critically for important intellectual content and played a major role in the implementation of the whole surveillance system. All authors contributed to the article and approved the submitted version.

## Conflict of Interest

The authors declare that the research was conducted in the absence of any commercial or financial relationships that could be construed as a potential conflict of interest.

## Publisher's Note

All claims expressed in this article are solely those of the authors and do not necessarily represent those of their affiliated organizations, or those of the publisher, the editors and the reviewers. Any product that may be evaluated in this article, or claim that may be made by its manufacturer, is not guaranteed or endorsed by the publisher.
